# Programmable thermal emissivity structures based on bioinspired self-shape materials

**DOI:** 10.1038/srep17682

**Published:** 2015-12-04

**Authors:** N. Athanasopoulos, N. J. Siakavellas

**Affiliations:** 1 Department of Mechanical Engineering & Aeronautics, University of Patras, 26500, Patras, Greece

## Abstract

Programmable thermal emissivity structures based on the bioinspired self-shape anisotropic materials were developed at macro-scale, and further studied theoretically at smaller scale. We study a novel concept, incorporating materials that are capable of transforming their shape via microstructural rearrangements under temperature stimuli, while avoiding the use of exotic shape memory materials or complex micro-mechanisms. Thus, programmed thermal emissivity behaviour of a surface is achievable. The self-shape structure reacts according to the temperature of the surrounding environment or the radiative heat flux. A surface which incorporates self-shape structures can be designed to quickly absorb radiative heat energy at low temperature levels, but is simultaneously capable of passively controlling its maximum temperature in order to prevent overheating. It resembles a “game” of colours, where two or more materials coexist with different values of thermal emissivity/ absorptivity/ reflectivity. The transformation of the structure conceals or reveals one of the materials, creating a surface with programmable – and therefore, variable- effective thermal emissivity. Variable thermal emissivity surfaces may be developed with a total hemispherical emissivity ratio (εEff_H/εEff_L) equal to 28.

Thermal management is one of the most significant and discussed disciplines in applied physics and engineering. Conductive heat flux and thermal radiation manipulation could lead to efficient thermal systems and passive thermal control. Regarding radiative heat transfer, variable thermal emissivity materials and artificial structures (microelectromechanical systems) have been studied and developed using different approaches in order to control the absorption, reflection and the rejection of heat. There are two parameters which are responsible for the radiative behaviour of a body; i) the radiative properties, such as emissivity coefficient (ε), absorptivity (a), reflectivity (ρ), and transmissivity (τ) of the body’s material, and ii) the view factor of the body’s geometry (F).

Variable emissivity/absorptivity structures have been developed and studied, leading to the realization of very complex, heavy mechanical devices such as louvers^1^ as well as microelectromechanical systems (MEMs)^2^,^3^,^4^, which incorporate hinges and actuation mechanisms. These complex microstructures, such as micro-machined louvers or shutter arrays^2^,^4^, have been suggested in order to achieve thermal control by varying the effective thermal emissivity of a surface. The microshutter arrays consist of different parts and can be actively actuated by small electrostatic comb drive motors which provide linear control of the effective thermal emissivity with a turndown ratio equal to 5 (maximum emitted energy/minimum emitted energy)^4^. Furthermore, the ability of the electrochromic materials to change their reflectance in the infrared wavelength via an applied bias voltage, is another promising approach. Very recently, researchers found that the thermal emissivity of vanadium dioxide (VO_2_) presents an anomalous thermal emittance profile which is strongly correlated with temperature^5^. Moreover, electrically switchable broadband infrared reflectors have been fabricated, using polymer stabilised cholesteric liquid crystals. The IR reflectors can change their reflection/transmission properties through the application of an electrical voltage^6^.

The current study aims at developing a new generation of adaptable structures with variable and programmable thermal radiative properties. Our inspiration is drawn from Nature, where certain plants change their shape when in need to absorb more or avoid excess sunlight. While the extremely heavy variable emissivity mechanical systems (louvers) and complex MEMs use hinges, actuators in order to develop simple movements, in Nature extremely complex movements can be realized through the materials’ self-shaping & self-folding capabilities in response to a stimulus. Thermonastic movements, meaning ‘folding caused by a temperature stimulus’, are realized in order to transform the shape of leaves/petals under a temperature stimulus. The view factor (geometrical characteristics of the radiative object), and the material that is exposed to the environment are regulated in Nature for prevention from overheating^7^,^8^. More specifically, the drooping of rhododendron leaves protects them from damage due to high irradiance and cold temperatures^8^, while poplar’s leaves present dual thermal reflectivity values on both of their leaves’ surfaces for damage prevention^7^.

Following that concept and taking it one step further, the scientific breakthrough is the study and development of materials and structures which are a priori fashioned to passively respond to temperature to which they are exposed, by changing their geometry and their material thus either absorbing or reflecting sunlight accordingly. It resembles a “game” of colours, where two or more materials of different thermal radiation values (emissivity/absorptivity/reflectivity) coexist. The surfaces which would consist of a plethora of unit-cell structures, could serve as adaptable ‘skins’ and can be placed on top of any surface in order to passively control its temperature. More specifically, a “forest” of small structures opens and closes in a fashion similar to the flower petals during the day-night cycle.

A schematic diagram of the structure employed in this study is illustrated in Fig. 1. When the structures are open, a material of certain thermal radiative properties (e.g. black coating) is exposed to the environment, Fig. 1b, whereas when closed, a material of different properties (e.g. Aluminium coating) is exposed, Fig. 1c. In this way, it is possible to fashion surfaces consisting of a plethora of small structures (arrays), a priori tuned to passively respond to predetermined temperature values by absorbing/emitting or reflecting thermal radiation, Fig. 1a. Therefore, the self-shape structures can behave as absorbers/emitters when exposed to certain temperature values, as well as “perfect” reflectors to others.

In order for these complex movements to be realized, it is necessary that materials of two-way shape memory be developed. Materials that are capable of changing their shape are the well-known shape memory alloys (SMAs)^9^ and the exotic shape memory polymers (SMPs)^10^,^11^,^12^, as well as temperature sensitive hydrogels^13^. The mechanism that transforms the geometry of SMPs is based on the phase change transition at molecular scale^10^, while SMAs transformation is based on the martensitic to austenitic phase change^9^.

In contrast, the dead tissues of various plants are designed to undergo predetermined changes according to changes in environmental conditions, through the triggering of the movement of the structure due to the fibrous anisotropic nature of the plant’s material^14^,^15^,^16^,^17^. The coefficients of hygroscopic and thermal expansion are the corresponding parameters characterizing such changes of physical dimensions of the plants’ dead tissues^14^,^15^,^16^,^17^. The coefficient of thermal expansion (CTE) mismatch between two fibrous anisotropic layers, creates shape memory materials which alter their shape via the developed internal stresses^17^,^18^. Thus, the self-shape materials can be triggered (start to transform themselves) by the temperature stimulus and through the large CTE of the anisotropic multilayer material. Actually, these materials act similarly to bi-metallic strips (bimorph metallic layers^18^), while maintaining anisotropic characteristics. Bimorph metallic layers^18^ can perform only bending movements, while anisotropic multilayer materials can perform bending, twisting or complex combined modes^17^,^18^. As a result inexpensive “shape memory materials” can be developed to passively react to a very broad range of thermal requirements (−200 °C to 350 °C), which cannot be achieved by SMPs and SMAs. The term self-shape refers to materials that have the ability to transform (morph) themselves due to the large hygroscopic expansion or CTE mismatch which manifests due to the anisotropic nature of the material’s micro-structure^17^,^18^.

This particular shape memory materials could be developed through the use of i) multi-layered fibrous anisotropic materials or ii) anisotropic nano-composites, iii) nano-reinforced multilayer hydrogels^16^ or iv) through the development of a particular micro-structure with anisotropic properties, similar to an anisotropic cellular material in multilayer form. The self-shape structure can be transformed under temperature stimulus, while the 1^st^ memory shape and the 2^nd^ memory shape can be determined by a) the geometry, b) the anisotropic nature of the material as well as c) the homogeneous or non-homogeneous nature of the material’s structure.

This study aims at revolutionizing the materials and structures in the frame of radiative heat management. The programmed behaviour can be achieved through the shaping transformation, by regulating the view factor and the material that is exposed to the environment. The transformation of the structure conceals or reveals one of the materials, creating a surface with tunable and programmable effective thermal emissivity (ε_eff_). Their radiative properties can be programmed through the study of the following parameters: i) the materials’ physical and mechanical characteristics, ii) the geometry of the self-shape structures, iii) their size, iv) the distance between the self-folding structures (in the case of array), Fig. 1a and finally v) the materials which alternate in exposure to the environment.

These tunable and programmable emissivity structures could find a plethora of applications in energy saving and control in many fields, varying from architecture and energy systems, to space applications where the minimal, multifunctional design and the weight limitations are crucial parameters.

The paper is organized as follows: The variable effective thermal emissivity mechanism of a self-shape unit cell and the theoretical modelling are described in the next. Then, the experimental results at macro-scale are shown, followed by some numerical results for structures at a smaller scale. In the experimental Section the materials and their preparation are described and, finally our conclusions are given.

## Activation of the self-shape material

### Variable effective thermal emissivity mechanism

Structures with variable emissivity, which have the ability to absorb or reflect and reject infrared radiation (or visible light) using specific programmed self-shape materials, are proposed and studied. The proposed self-shape structures are dynamic structures capable of reacting under temperature deviations, presenting variable and programmed thermal emissivity behaviour. The structure reacts according to the temperature of the surrounding environment, or the radiative heat flux. For example, a surface which incorporates self-shape structures can be designed to quickly absorb radiative heat energy at low temperature levels, but simultaneously is capable of passively controlling its maximum temperature in order to prevent overheating.

**Figure 1:**
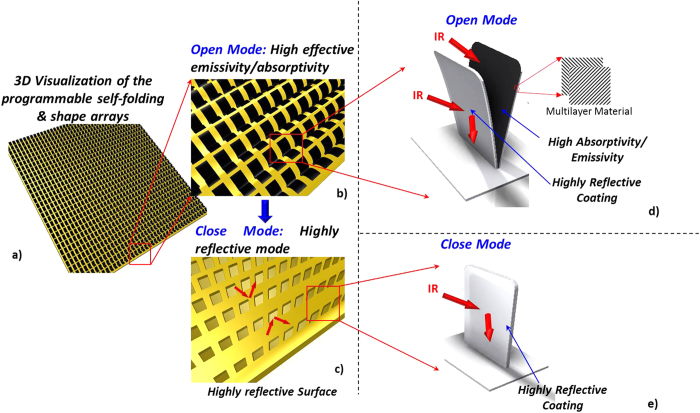
(**a**) 3D visualization of the programmable self-shape arrays, (**b**) open mode of the self-shape structures, (**c**) closed mode of the self-shape structures, (**d**) unit cell in open mode at temperature T_m1_, (**e**) unit cell in closed mode at temperature T_m2_.

**Figure 2:**
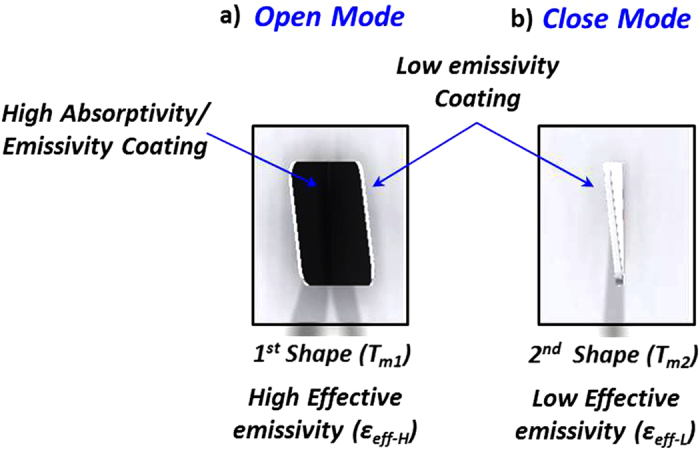
Plan view of the (a) Open mode of the self-shape structure (high effective emissivity), (b) Close mode of the self-shape structure (low effective emissivity).

A unit cell of a self-shape structure consists of a main surface and two thin (Fig. 1d) multilayered curved surfaces. The external surface is made of a low emissivity material (ε_L_), and the internal surface of the structure is made of a material of higher emissivity (ε_H_) Fig. 2. The self-shape structures have been placed on the top of the surface, and have been programmed to have a predictable specific geometry at an initial temperature (Tm_1_), which is, essentially, the first shape that has been memorized by the material itself, Figs. 1d and 2a. At a second temperature level (Tm_2_) (where Tm_2_ > Tm_1_) the structure has to memorise a second shape, Figs. 1e and 2b.

The material has a two-way memory, and the morphing mechanism is triggered by the CTE mismatch of the multilayer structure and the generated stresses in the layered material. The internal generated stresses force the structure to transform into a closed or open geometry, according to the developed temperature. In this way, the dimensions of the cavity are modified.

**Figure 3:**
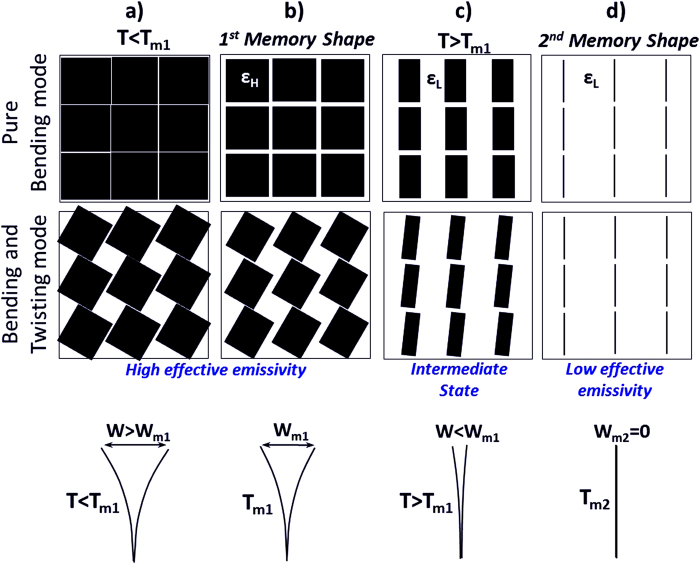
Plan view of the variable emissivity surface with self-shape structures which are under pure bending, or combined bending-twisting mode. (**a**) T < T_m1_ (ε > ε_eff_H_). (**b**) T = T_m1_ (ε = ε_eff-H_). (**c**) T > T_m1_ (ε < ε_eff-H_). (**d**) T = T_m2_ (ε = ε_eff-L_«ε_eff-H_).

Figure 3 shows a variable emissivity surface in pure bending mode, in combined bending- twisting mode and the corresponding gap as a function of temperature. The dimensions of the cavity are reduced as the temperature increases, due to the triggering of bending and twisting modes. The effective emissivity value (ε_eff-H_) of the self-shape material’s first shape (Fig. 3b) decreases as long as the temperature increases, and when the structure closes completely due to the 2^nd^ second memory temperature value (T_m2_), the value of the effective thermal emissivity reaches its minimum (ε_eff-L_) at the steady-state and the area of the cavity is nullified (Fig. 3d). The developed thermal stresses force the material (and the structure) to be in closed mode in higher temperatures, and the initial shape starts being recovered in temperatures lower than (Tm_2_).

The effective thermal emissivity of a surface can be programmed and regulated by taking into consideration the geometrical characteristics of the unit cell, the direction and the combination of the multilayer material, and all the physical and mechanical properties of the anisotropic material. Pure bending modes deform the material to an initial programmed shape at temperature Tm_1_ (Fig. 3b).

The effective emissivity takes a value that is related to: 1) the emissivity of the outer material (ε_L_), 2) the emissivity of the inner surface (ε_H_), and 3) the geometry of the unit cell which is a function of the modulus of elasticity at the two principal directions (E_1_, E_2_), Poisson ratios (ν_12_, ν_21_), shear modulus (G_12_), the direction of the fibres of each layer and the temperature. The adjacent ply of a different ply angle will restrict the adjoining ply in deforming freely. The residual stresses are set up in individual plies of a laminate as a result of the enforced common deformations, in which the plies are not allowed to individually deform freely. Thus, the gap (W) between the layers of the self-shape material is a function of temperature. When the surface rejects energy, the temperature of the surface decreases and the gap (W) increases (Fig. 3a) due to the internal developed stress and the microstructural rearrangement. On the other hand, when the surface absorbs energy, the temperature increases and the gap decreases (Fig. 3c) until it reaches a very small value (W_m2_ ≈ 0, Fig. 3d), leaving exposed only the material with the low emissivity value (ε_L_).

Three different approaches can be adopted, 1) materials that enable pure bending modes with curvatures (k_xx_ or k_yy_), 2) materials that enable pure twisting modes (k_xy_), and 3) materials that enable twisting and bending modes (k_xx_, k_yy_, k_xy_).

### Theoretical modelling

Fibrous composite laminates can be manufactured at temperatures higher than room temperature, and then are allowed to cool to ambient temperature. During the curing process of the polymeric resin after the cross-linking, the composite curing process takes place. The common strain can be expressed by Eq. 1 taking into consideration the classic mechanics of composite materials^19^.


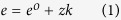


where (ε^ο^) is the membrane strain and (–zk) the bending strain. Taking into consideration the temperature parameter, Eq. 1 is transformed to Eq. 2.


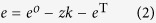


where (ε^Τ^) is the temperature induced deformation, and the multilayer material residual stress is equal to Eq. (3).


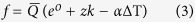


where (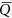) is the reduced stiffness matrix, (z) is the section thickness, (k) expresses the midplane curvatures, and (α), (ΔT) are the coefficient of thermal expansion term and the temperature difference respectively. The residual force intensity on the anisotropic layers is obtained by integrating the residual stress over the thickness of each layer. The residual moment intensity on the anisotropic layer is obtained by integrating the product of the residual stress and the moment arm (z), over the thickness of the total thickness of the multilayer anisotropic material. The developed force intensities and moments of the multilayer material can be expressed by Eqs 4 and 5.


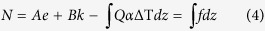



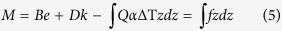


where (A) is the multilayer material’s membrane stiffness, (B) is the multilayer material’s coupling stiffness, and (N) is the equivalent laminate force intensities due to the free thermal strain effects. Similarly, the laminate moment intensities (M) can be expressed by Eq. 5, where (B) is the laminate coupling stiffness and (D) is the laminate bending stiffness. The membrane, coupling and bending stiffness matrices can be expressed by the set of Eq. 6.


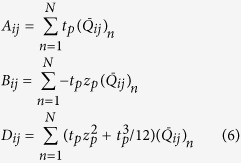


The governing equations, in matrix form, can be expressed by the following Eq. 7.


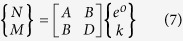


Solving a system of linear equations the strain values (e_x_, e_y_, e_xy_), the curvatures (k_xx_, k_yy_, k_xy_) and consequently the final shape of the self-shaped structure can be predicted. Furthermore, the dimension of the gap (W), Fig. 3 can be calculated using all the geometrical characteristics of the self-shape structure (height, width) as a function of temperature. The out of plane displacement (W/2) can be predicted integrating over the calculated curvatures through the Eq. 8.


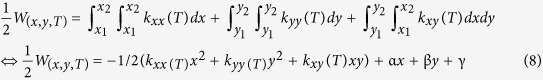


The area of the resulted high emissivity gap (A_(T)_ = 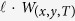) can be calculated according to Eq. 8, where 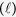 is the width of the unit cell and (α, β, γ) can be determined taking into consideration particular mechanical boundary conditions. Knowing all the materials’ geometrical characteristics and then, calculating the curvatures as a function of temperature, the high and low emissivity regions can be accurately determined, while the temperature of the self-shape structure can be determined as a function of the radiation heat flux intensity of the heat source. The parameters of interest, i.e. radiosity, emissivity, temperature of each surface, as well as the irradiation, coming either from other surfaces or from external radiation sources, are related by the following equations:

The radiosity leaving a surface is defined by equation (9) as the radiation energy leaving a surface per unit area per unit time (W/m^2^):


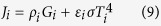


where (*ρ*_*i*_) and (*ε*_*i*_) are the surface reflectivity and emissivity respectively, while (i) denotes the surface of the low or the high emissivity material.

The irradiation, (G), of the surface can in general be written as a sum according to:


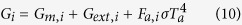


where (*G*_*m*_) is the mutual irradiation, coming from other boundaries, (*G*_*ext*_) is the irradiation from external radiation sources, (*F*_*a*_) is an ambient view factor whose value is equal to the fraction of the field of view that is not covered by other boundaries, while (*T*_*a*_) is the assumed far-away temperature in the directions included in (*F*_*a*_). The radiosity, in turn, is a function of (*G*_m_). In fact, (*G*_*m*_) is the integral over all visible points of a differential view factor (F) times the radiosity (J) of the corresponding source point. In the discrete model, (*G*_*m*_) may be expressed as the product of a view factor matrix and a radiosity vector. Inserting now the Eq. 10 for (*G*) in Eq. 9, the following equation is obtained:


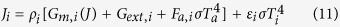


Assuming an ideal gray-body, the last equation becomes:


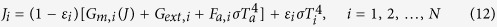


Equation (12) results in a linear equation system in (*J)* that is solved in parallel with the equation for the temperature, (*T)*, while (N) expresses the number of surfaces that the overall structure consists of. Each surface (i) is characterized by its own temperature and emissivity, reflectivity values.

## Results and Discussion

### Experimental results in macro-scale

Macro-scale self-shape structures were developed in order to study the absorption of heat flux radiation, using self-shape materials. The self-shape material consists of two layers (125 μm thickness) of a carbon fiber/epoxy composite, and an aluminium foil layer (20 μm thickness) with the following stacking sequence (black coating/0/90/aluminium foil). The final morphed shape of the structure is absolutely flat at a temperature equal to Tm_2_ ≈ 145 °C, without external constraints.

According to Eq. 7 the calculated curvatures are the following: k_xx_ = 9.76 × 10^−3^ mm^−1^, k_yy_ = −9.76 × 10^−3^ mm^−1^ and k_xy_ = 0. The structure is fully closed at approximately T ≥ 145 °C, while the emissivity of the outer surface and the inner surface is equal to ε_L_ = 0.08 and ε_H_ = 0.93 respectively.

**Figure 4:**
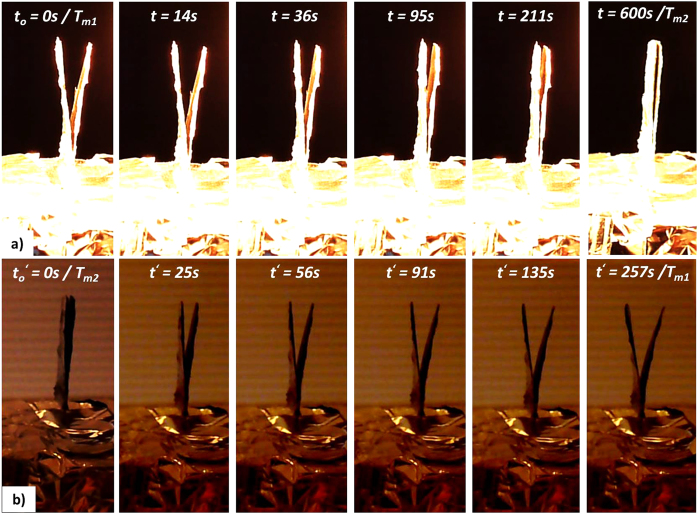
Iso-view of the variable emittance self-shape structure (Tm_1_ ≈ 20 °C, Tm_2_ = 145 °C). (**a**) Absorption of energy during heating stage. (**b**) Rejection of energy during cooling stage.

The self-shape structure bends around the x-axes, reaching a steady-state temperature and shape at a specific radiative heat flux, Fig. 4. After the surface reaches the steady state temperature, the shape remains deformed and constant. Any deviation of the temperature field leads to a different shape. The initial gap (W_m1_) of the self-shape structure, using the aforementioned multilayer material, was 9.2 mm.

Figure 4a presents the transient response of the self-shape structure during the heating stage, using 500W lamps above the self-shape structure. For time values above 600s, the self-shape structure almost reaches the second memory shape at T_m2_ and remains steady. Immediately after removing the radiative heat source, the structure seems to start recovering its initial shape, Fig. 4b.

**Figure 5:**
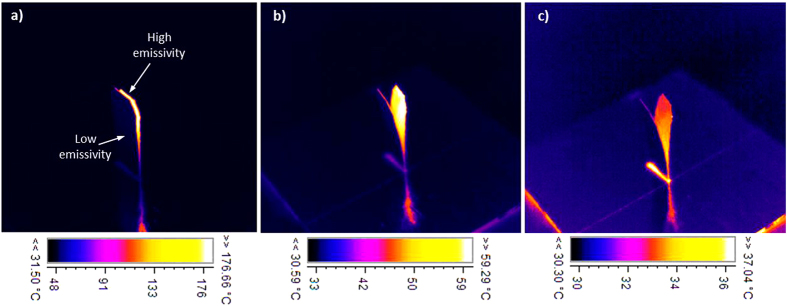
Iso-view of the thermal camera recorded image during the cooling stage of the self-shape structure (Tm_1_ ≈ 20 °C, Tm_2_ = 145 °C). (**a**) T > Tm_2_. (**b**) T < Tm_2_. (**c**) T ≈ Tm_1_.

Figure 5 presents the thermal camera snapshots during cooling stage of the self-shape material. The emissivity coefficient of the thermal camera has been set to be equal to ε_therm_ = 0.93. Due to the fact that the cavity has a thermal emissivity coefficient equal to the set point emissivity of the thermal camera, the recorded temperature is the actual temperature of the self-shape structure while the temperature of the outer side, which consists of the low emissivity material, seems to be equal to 30–35 °C. It is obvious that this measurement is incorrect due to the different emissivity that has been set to the thermal camera. Figure 5 prooves that during temperature increase, the emitted/absorbed thermal radiation decreases simultaneously because of the gap reduction due to the elimination of the gap area A_(T)_. Moreover, the temperature field of a self-shape structure with 1^st^ shape memory temperature T_m1_ = 25 °C and a 2^nd^ shape memory temperature T_m2_ = 230 °C is presented in the supplementary information section during the heating and cooling stage.

In order to measure the temperature increase rate, two thermocouples have been placed on the bottom of the self-shape structure below a thin copper substrate. Three different materials were developed and tested (Material 1, Material 2, Material 3). The internal coating has an emissivity and absorptivity approximately equal to 0.93, while the external aluminium film has emissivity value equal to 0.08, while the absorptivity value is equal to 0.15.

**Figure 6:**
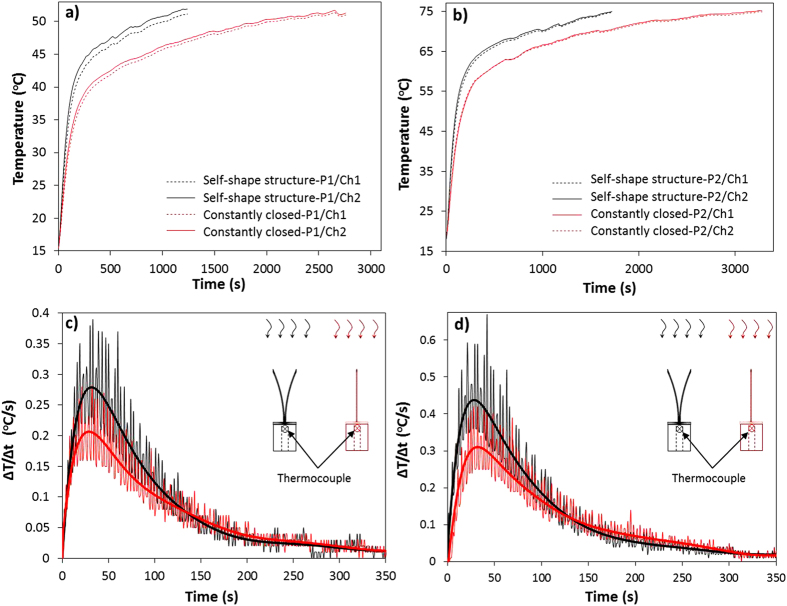
(**a**,**b**) Comparison of the measured temperature as a function of time for the self-shape structure and the closed structure for two different power levels. (**c**,**d**) Temperature increase rate as a function of time (ΔΤ/Δt (t)). (Tm_1_ ≈ 20 °C, Tm_2_ = 145 °C).

**Figure 7:**
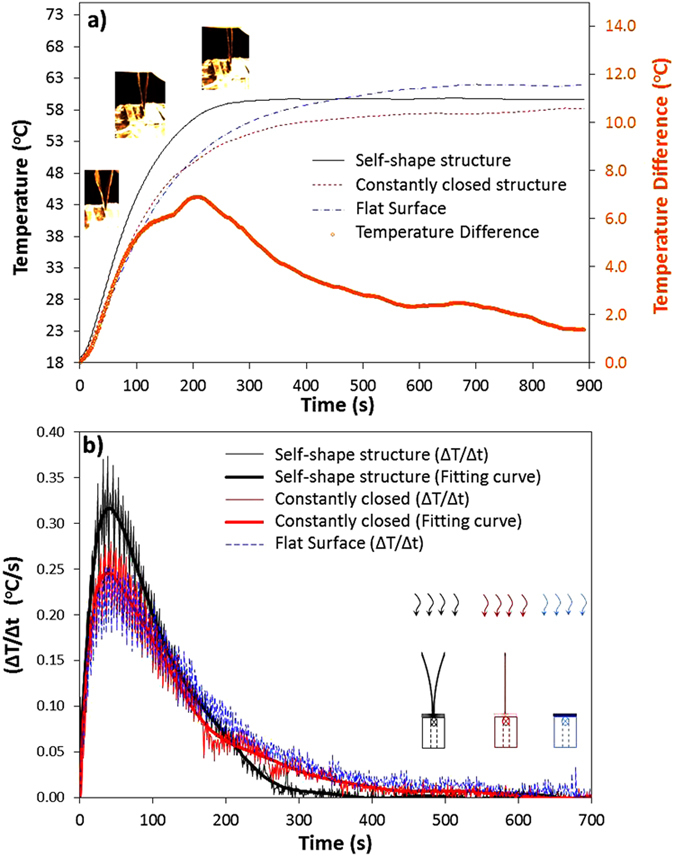
(**a**) Temperature as a function of time for the three examined cases. (**b**) Temperature increase rate as a function of time (ΔΤ/Δt (t)). (Tm_1_ ≈ 20 °C, Tm_2_ = 85 °C).

The recorded temperature profile of the self-shape structure was compared to:

1. A unit cell which is completely closed, where the emissivity of the material is approximately equal to ε = 0.08 (detail of Fig. 6).
2. A flat surface with thermal emissivity approximately equal to 0.08 (detail of Fig. 7).

### Material 1 (Tm_1_ ≈ 20 °C, Tm_2_ = 145 °C)

Figures 6a,b present the temperature as a function of time for two different radiative power levels (P1 = 250W and P2 = 500W). Both experiments present that the heat absorption of the self-shape structure is faster than in the case of the constatly closed structure. As it is clearly illustrated, 1100 s are required in order to heat up the self-shape structure to 53 °C, while the closed structure strucutre needs 2800 s to heat up to the same temperature. Observing Fig. 6a, the self-shape strucuture absorbs energy 2.55 times faster than the closed strucuture. In the case that heat flux intensity is higher (Fig. 6b), the self-shape structure is transformed faster.

As a result, the only surface that is left exposed to the environement is the one with the low emissivity (ε_L_ = 0.08). In Figs 6c,d, the temperature incease rate (ΔΤ/Δt (t)) is presented for the two cases. A polyonimial fitting curve was calculated in order to integrate below the (ΔΤ/Δt (t)) curve, and to calculate the absorbed energy in any case. Comparing the heating rates, it is clearly observed that the heating rate of the self-shape structure at the 1^st^ power level is 37.5% higher at the biggining of the phenomenon. At the second power level the heating rate is 35.5% higher than that of the constantly closed structure.

The self-shape structure occupies 80% of the total exposed area at environmental temperature, while after 600s only 2−3% of the black coating is exposed to the atmosphere (closed mode).

### Material 2 (Tm_1_ ≈ 20 °C, Tm_2_ = 85 °C)

A second series of measurements were completed, which concluded to solid results using different composite materials (T_m2_ = 85 °C). The heat absorption of the self-shape structure was compared to a flat surface, which had the same area and thermal emissivity, equal to ε_flat_ = 0.08. Figure 7a presents the recorded temperature as a function of time for the self-shape structure, the constantly closed structure and the flat surface.

According to Fig. 7b, the temperature increase rate (ΔΤ/Δt) is presented for the three cases. The self-shape structure absorbs the radiative heat energy faster than the other two cases. Furthermore, we observe that the temperature difference (bold black line) between the self-shape structure and the constantly closed structure increases up to 7 °C and decreases to 1.3 °C, when the self-shape structure is at the 2^nd^ memory shape.

Comparing again the heating rates, it can be clearly observed that the heating rate of the self-shape structure at the 1^st^ power level is 30% higher at the biggining of the phenomenon.

### Material 3 (Tm_1_ ≈ 20 °C, Tm_2_ = 230 °C)

**Figure 8:**
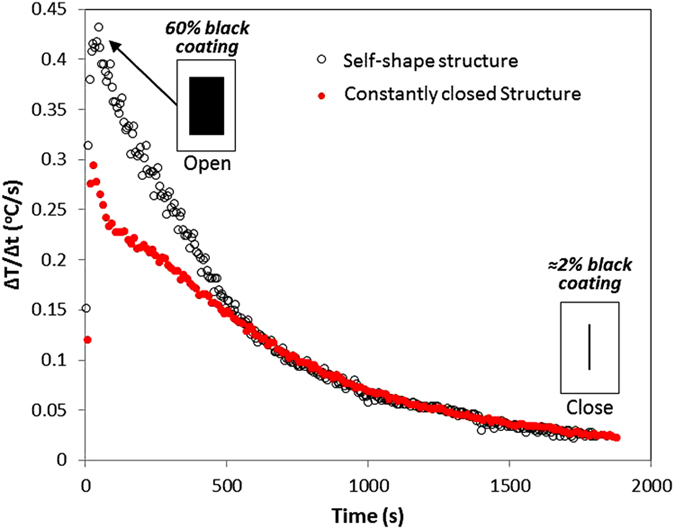
Temperature increase rate as a function of time in vacuum environment. (Tm1 ≈ 20 °C, Tm2 = 230 °C).

Figure 8 presents preliminary experimental measurements in vacuum environment using a similar setup (see experimental section) under vacuum environment. The vacuum level was deviated between 3–5 Pa. The self-shape structure occupies 60% of the total exposed area at environmental temperature, while after 1000s only 2% of the black coating is exposed to the atmosphere (closed mode). The temperature increase rate at the beginning of the phenomenon is 49% higher in comparison to the constantly closed structure. If the self-shape’s gap occupied a larger percentage of the surface, then the temperature increase rate would be higher.

### Numerical modelling of self-shape structures at smaller scale

The self-shape structures at macroscale are capable of changing their thermal radiation properties according to temperature, but the changing span of the radiative properties is much less than the respective for self-shape structures at micro-scale. The development of these self-folding arrays at microscale is very crucial due to the fact that the efficiency of these programmable thermal radiation materials is related to the length scale of these unit cells. Preliminary numerical calculations prove that the development of micro-structures can theoretically allow the development of materials capable of extremely changing their emissivity or absorptivity value from 0.95 to 0.03 for a specific temperature differential (ΔΤ). Therefore, the desirable ratios emissivity and absorptivity values as a function of temperature can be fashioned through such materials.

The efficiency of the self-shape materials to absorb or reflect the radiative heat flux, was examined in micro-scale using numerical models. The effective thermal emissivity as a function of temperature was studied. A similar self-shape structure with dimensions of (700 μm height, W = 250 μm and 25 μm thickness) was studied, and a coupled multiphysics problem was solved using COMSOL software. The input is the radiative heat flux above the structure, and the output is the displacement of the tip and the temperature at the steady state. Three different geometries were examined in order to compare the results and extract the effective thermal emissivity of the self-shape structure: a) self-shape structure (ε_H_ = 0.96, ε_L_ = 0.025), b) the structure which is constantly in closed-geometry mode (ε_L_ = 0.025), and the c) flat surface (ε_L_ = 0.025). Polished gold and silver may present extremely low emissivity values. For this reason we may assume that using a first material with high emissivity values and a second material with extremely low emissivity values, a self-shape structure with tuned radiative properties can be developed having very high ratios (ε_eff_H_/ε_eff_L_, α_eff_H_/α_eff_H_).

**Figure 9:**
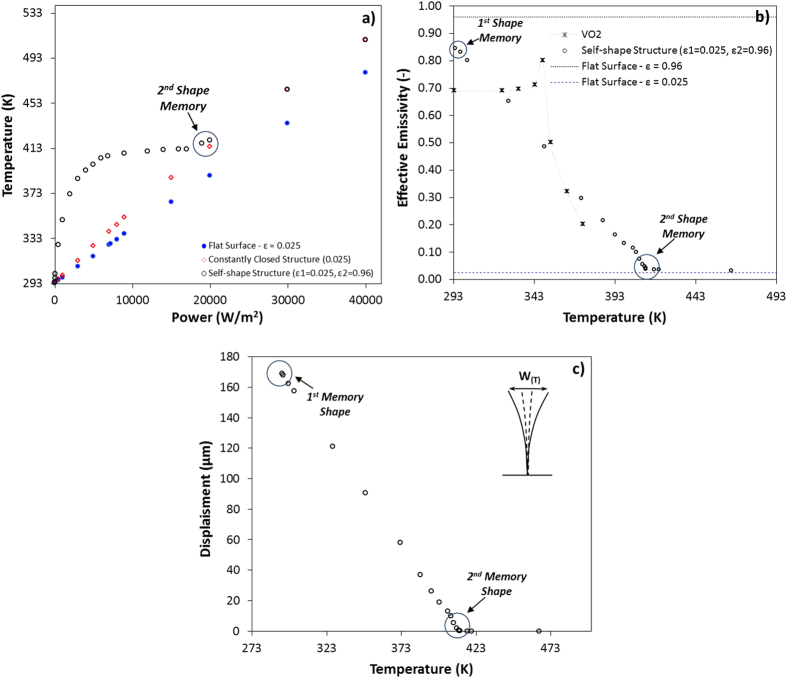
Theoretical calculations using metaphysics modelling with COMSOL software for a self-shape structure of 700 μm height, (0/90/gold foil). (**a**) Calculated temperature as a function of radiative heat flux. (**b**) Calculated effective emissivity of the self-shape structure. (**c**) Gap (W) as a function of temperature at the tip of the structure.

Figure 9a presents the calculated temperature as a function of heat flux. The flat low emissivity structure is illustrated by the blue dots, which have a predictable behaviour, as well as the constantly closed structure which is illustrated by the red dots. The self-shape structure presents an anomalous behaviour due to the transformation of the structure. The view factor changes, as well as the ratio of the high and the low emissivity areas. This non-canonical behaviour of the heat-flux vs the temperature curve of self-shape structure can be clarified by observing the effective emissivity vs the temperature curve. The effective emissivity of the self-shape structure presents a linear behaviour as function of the developed temperature, with two discrete boundaries. The effective emissivity of the material takes values between 0.85 and 0.03. This leads to a ratio (εEff_H/εEff_L) equal to 28.

Through Finite Element Modelling (FEM) it becomes possible to predict the shapes and dimensions of the self-folding unit cells which are capable of extremely changing their geometry and their radiative properties, or are capable of following specific temperature profiles (a priori temperature dependency). The energy absorbance is faster at lower temperatures, while after a first critical point the absorbance remains constant. At a second critical point, the self-shape structure is completely closed, and the high emissivity area is nullified. This critical point is presented at the second memory shape of the self-shape structure. After this point, the energy absorbance is equal to the energy absorbance of the completely closed structure. A brief comparison between the variation of the VO_2_ emissivity and the self-shape structure is presented at Fig. 9b, revealing the performance of the self-shape materials, while Fig. 9c presents the out-of plane displacement (W) of the edge of the self-shape structure as a function of temperature. As it can be observed from the graphs, a programmed emissivity can be achieved using self-shape structures in order to absorb and reflect different amounts of energy at different temperature levels. One step further is the development of materials which can be programmed, in order to achieve emissivity profiles that deviate between two values, ultimately reaching a minimum at a specific temperature. For temperatures higher than this, the emissivity increases linearly or non-linearly as the temperature increases.

## Conclusions and Discussion

Bioinspired approaches may mimic the behaviour or structural designs in micro and macro-scale from nature in order to develop future materials and structures for optimized thermal applications in Architecture, Space and Energy systems.

The variable emissivity of these two-way shape memory structures and its temperature dependency can be adopted in a very broad range of thermal requirements, something which greatly increases a thermal engineer’s design flexibility. Use of bioinspired self-shape materials is a pathway for the development of low cost and lightweight thermal materials and structures, capable of conducting very complex movements and of passively reacting to temperature. These are extremely difficult to engineer through conventional design approaches, whereas the effective emissivity vs temperature could be programmed according to specific profiles.

Programmable variable thermal emissivity structures were developed using four layers (1^st^ coating/layer in θ_1_ direction/layer in θ_2_ direction/2^nd^ coating) and studied thoroughly in macro-scale, proving that these passive structures can be used in order to reflect or absorb infrared radiation as function of temperature. Regarding the experimental results, the heat emission/absorption was 3 times faster with the use of the aforementioned materials and geometries, while the maximum temperature of the structure was passively regulated. On the other hand, the theoretical calculations demonstrate that an emissivity ratio equal to 28 is feasible through the implementation of the same self-shape materials at smaller scale.

Moreover, artificially created anisotropic behaviour can be achieved in order to respond to directional radiation stimuli through the arrangement and the direction of the self-shape unit cells. Finally, the absorbed or reflected wavelengths may also be tuned.

## Experimental Section

### Materials and preparation

Different composite materials were used in order to develop self-shape structures at macro-scale. The following materials were used in order to measure the temperature increase rate in Figs 6 and 7. The carbon fiber composite multilayer anisotropic material was fabricated with Sigrafill/Sigratex UD-prepreg, 242 g/m^2^ using the autoclave manufacturing process. The applied pressure at the manufacturing stage of the CFRP laminates was (P = 0.8 MPa). The curing cycle of the material was 2.5 h at ≈145 °C. The second structure was fabricated using a T300 unidirectional inlay with a common epoxy resin system L1100, and the infusion process was carried out at a pressure value of P = 0.1MPa. The curing cycle of the material was 8h at 80−85 °C.

**Figure 10:**
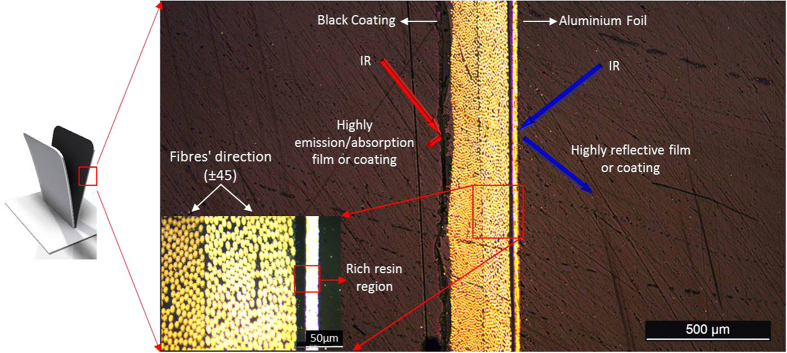
Cross section of the composite material. The thin aluminium foil and the black coating can be observed at the right and left side of the cross section respectively.

Figure 10 presents an optical microscope image of the cross section of the materials at (±45°), as well as a magnified image. The 1^st^ layer is a black coating, the 2^nd^ layer is a carbon fiber composite layer at −45 degrees direction, the 3^rd^ is another carbon fiber composite layer at 45 degrees direction and the 4^th^ layer is an 20 μm aluminium film. The thermal radiation is reflected by the aluminium foil side and is absorbed by the left side of the developed material.

### Measurement Setup

**Figure 11:**
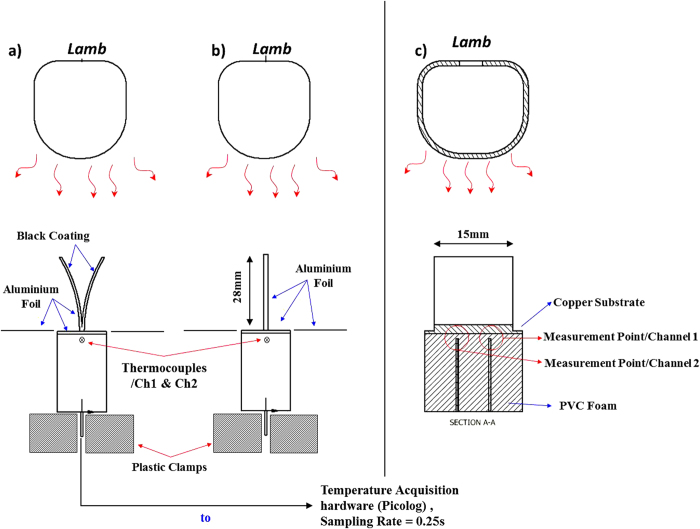
Experimental measurement setup. (**a**) open mode, (**b**) closed mode, (**c**) cross section of the structure.

In order to measure the temperature increase rate, two thermocouples have been placed (Channel 1 & Channel 2) at the bottom of the self-shape structure below (under the copper substrate, Fig. 11c). At the beginning of the heating stage, the unit cell is open, Fig. 11a. A temperature acquisition device (Picolog TC-08) was used in order to continuously record the temperature (sampling rate = 0.25s) until the system reached its steady state value, Fig. 11b. The power level of the lamp was regulated using an AC variable transformer. The flat substrate of the unit cell has been made by 0.5 mm copper plate. Low thermally conductive PVC foam was attached, as well as plastic clamps in order to hold the thermocouples and nullify the heat transfer from the sides of the block. A high resolution thermal camera (Flir SC660) was used in order to record the temperature field in each case.

## Additional Information

**How to cite this article**: Athanasopoulos, N. and Siakavellas, N. J. Programmable thermal emissivity structures based on bioinspired self-shape materials. *Sci. Rep.* **5**, 17682; doi: 10.1038/srep17682 (2015).

## Supplementary Material

Supplementary Information

Supplementary video 1
