# Supplementary material for: Programmable thermal emissivity structures based on bioinspired self-shape materials
Source: Sci Rep. 2015 Dec 4;5:17682. doi: 10.1038/srep17682 (PMC4669499; doi:10.1038/srep17682)
Supplement: Supplementary Information [file srep17682-s1.pdf]

Programmable thermal emissivity structures based on bioinspired self-shape materials

N. Athanasopoulos*[1], N. J. Siakavellas
Department of Mechanical Engineering & Aeronautics, University of Patras, 26500, Patras, Greece

Supplementary Information (Video file)

Temperature field (using Flir SC660 thermal camera) of a self-shape structure with 1st shape memory temperature at T_{m1} =25°C and a 2nd shape memory temperature at T_{m2} =230°C is presented in the following link during the heating and cooling stage.

[1] Corresponding author. Tel.:+302610997898;fax:+302610997241. E-mail address: nathan@mech.upatras.gr (N. Athanasopoulos).
